# Discordance of PIK3CA and TP53 mutations between breast cancer brain metastases and matched primary tumors

**DOI:** 10.1038/s41598-021-02903-x

**Published:** 2021-12-07

**Authors:** Anna Thulin, Carola Andersson, Elisabeth Werner Rönnerman, Shahin De Lara, Chaido Chamalidou, Arnd Schoenfeld, Anikó Kovács, Henrik Fagman, Fredrik Enlund, Barbro K. Linderholm

**Affiliations:** 1 Department of Oncology at Gothenburg University, Sahlgrenska Academy and University Hospital, 413 45 Gothenburg, Sweden; 2 Department of Oncology, Sahlgrenska University Hospital, Gothenburg, Sweden; 3 Diagnostic Center, Kalmar, Regional Hospital of Kalmar County, Kalmar, Sweden; 4 Department of Clinical Pathology, Sahlgrenska University Hospital, Gothenburg, Sweden; 5 Department of Oncology, SKAS, Skövde, Sweden; 6 Department of Pathology, Norra Älvsborg Hospital, Trollhättan, Sweden; 7 Department of Laboratory Medicine, Sahlgrenska Cancer Center, Institute of Biomedicine, Sahlgrenska Academy at University of Gothenburg, Gothenburg, Sweden

**Keywords:** Cancer, Genetics

## Abstract

There is limited knowledge of the biology of breast cancer (BC) brain metastasis (BM). We primarily aimed to determine the mutations in BCBM and to compare the mutational pattern with the matched primary breast cancer (BC). Secondary aims were to determine mutations in each subgroup (Luminal A-/B-like, HER2+ and TNBC) of BCBM, and to determine survival according to specific mutations. We investigated 57 BCBMs, including 46 cases with matched primary tumors (PT) by targeted Next Generation Sequencing (NGS) using the Cancer Hotspot Panel v2 (ThermoFisher Scientific) covering 207 targeted regions in 50 cancer related genes. Subtype according to immunohistochemistry was re-evaluated. NGS results fulfilling sequencing quality criteria were obtained from 52 BM and 41 PT, out of which 37 were matched pairs. Pathogenic mutations were detected in 66% of PTs (27/41), and 62% of BMs (32/52). *TP53* mutations were most frequent; 49% (20/41) of PTs and 48% (25/52) in BMs, followed by *PIK3CA* mutations; 22% (9/42) in PTs and 25% (13/52) in BMs. Mutations in *CDH1, EGFR*, *HRAS, RB1 CDKN2A* and *PTEN* were detected in single pairs or single samples. Mutational pattern was discordant in 24% of matched pairs. We show a discordance of *PIK3CA* and *TP53* mutations of roughly 25% indicating the need to develop methods to assess mutational status in brain metastasis where analysis of cell-free DNA from cerebrospinal fluid (CSF) has shown promising results.

## Introduction

Despite increasingly effective treatment, about 20% of patients with primary breast cancer (BC) suffer from metastatic disease and 15–40% of these patients eventually develop brain metastases (BM)^1^. Due to limited treatment options, and debilitating symptoms that greatly affect quality of life, BM is a dreaded outcome^2^. There is a large variation in survival after diagnosis of BM that ranges from Triple Negative Breast Cancer (TNBC), with a median survival of 4–5 months, to Human Epidermal growth factor Receptor 2 (HER2) positive (HER2+) BC with a median survival following BM diagnosis of 9–16 months^3,4^. The subtype of BC influences the risk of developing BMs and the BM free interval, with significantly higher incidence and shorter interval from recurrence to diagnosis of BM for patients with TNBC and HER2+ as compared to Luminal A-/B-like tumors^5,6^.

Local treatment such as surgery and/ or radiotherapy forms the basis of BM treatment. There is also clear evidence showing systemic therapies to have an effect on BM in patients ineligible for local treatment^7,8^. However, the blood–brain-barrier as well as the blood–tumor-barrier may hinder the passage of systemic therapies to the central nervous system, potentially hampering the effect of systemic treatment on BMs^9^. Another clinical problem is the difficulty to access metastatic tissue from the brain for genetic and immunohistochemical (IHC) characterization. Repeated studies have shown a discordance in the expression of steroid receptor and HER2 status between metastatic lesions and the primary BC^10^. A rapid development of new compounds targeting HER, CDK4/6, PARP and PIK3CA is underway. Hence, the need for analysis of BC tissue in the metastatic setting is apparent.

BM have become increasingly common in BC^6,11^, consequently, there is need for adequate information regarding the evolution of mutations, and histology in the metastatic process for correct therapy decisions^12–14^. There are studies investigating gene expression in metastatic lesions^15^. However, due to difficulties to sample tissue, BMs represent very few of investigated metastatic lesions.

We primarily aimed to determine the mutational status of key driver genes in BCBMs and to compare the results with mutations in matched primary BC using next generation sequencing (NGS). Secondly, we aimed to investigate the mutations in each BC subgroup (Luminal A-/B-like, HER2+ and TNBC) and to determine survival according to specific mutations.

## Materials and methods

### Patients

From hospital records of all diagnostic codes, patients with BMs from BC between 1994 and 2014 were identified. Patients received treatment of the primary BC four hospitals in the western region of Sweden. The diagnoses were confirmed in the patient’s charts and patients with available FFPE tissue from BMs were selected. Clinical characteristics, type of metastasis, progression, and survival were extracted from patient charts. PTs and BM tissue were evaluated, as specified below, when sufficient material was available. Data from the original report was utilized if deemed appropriated by the responsible breast pathologist if there was insufficient material available for re-evaluation by IHC. The study was conducted in accordance with the Declaration of Helsinki and the Sahlgrenska University Hospital Ethical Review Board; Gothenburg, Sweden approved the study (460-09, T592-14). Approval for the chart review and bio bank extractions was granted from each head of the participating departments.

### Immunohistochemistry

The available material was re-evaluated for histological type, nuclear grade, and receptor status. ER/PR, Ki67 and HER2 (HercepTest) IHC status was found as per standard procedures using the Dako Autostainer Link and the EnVision™ FLEX detection systems according to the manufacturer’s instructions. HercepTest was followed by SISH when the IHC was judged as 2+ or 3+. IHC was used to construct the subgroups. Luminal A-like was defined as ER positive, PR present and/ or low Ki67 (< 14%). Luminal B-like was defined as ER positive, PR absent and/or high Ki67 (> 15%). HER2+/ER+ was defined as amplified HER2 and ER positive. HER2+/ER− was defined as HER2 amplified and ER negative. TNBC was defined as lacking HER2 amplification, ER positivity and PR positivity.

### Next generation sequencing

#### Preparation of sample library and next generation sequencing (NGS)

DNA isolation from FFPE sections containing a minimum of 25% neoplastic cells as assessed by breast pathologists was performed using the QIAamp DNA FFPE tissue kit (Qiagen Gmbh, Hilden, Germany). DNA concentration was determined using NanoDrop™ 3300 with Quant-IT Picogreen dsDNA assay kit (ThermoFisher Scientific, Waltham, MA, USA). Ten ng of DNA was used to prepare barcoded libraries with the Ion AmpliSeq™ Library kit 2.0 (ThermoFisher Scientific, Waltham, MA, USA). The Cancer Hotspot Panel v2 (ThermoFisher Scientific, Waltham, MA, USA) covering 207 targeted regions in 50 cancer related genes was used (https://tools.lifetechnologies.com/content/sfs/brochures/Ion-AmpliSeq-Cancer-Hotspot-Panel-Flyer.pdf). Template preparation and enrichment was performed with the IonChef™ Instrument (ThermoFisher Scientific, Waltham, MA, USA). Eight barcoded samples were pooled per Ion 318™ v2 BC chip and sequenced on the Ion PGM™ System (ThermoFisher Scientific, Waltham, MA, USA). All steps were performed according to the manufacturer’s instruction. Matched germline DNA from the patients was not available in this retrospective study.

#### Data processing

After alignment to the hg19 human reference genome, variant calling by the Torrent Suite Software™ v.4.2.1.0 (ThermoFisher Scientific, Waltham, MA, USA) with subsequent manual filtering of known SNPs. Variants were visually inspected with the Integrative Genomics Viewer (IGV; Broad Institute, Cambridge, MA, USA). Mutations were manually curated as pathogenic, likely pathogenic, variants of unknown significance or as benign. For ten samples, NGS results could not be obtained due to insufficient quality of DNA. For individual variants the minimum accepted read depth was 500 and if the read depth was < 1000, an allele frequency of > 5% was required for positive variant calling.

### Statistical methods

Tests of differences between subgroups and mutations was performed using Chi-square test or Fischer Exact test, depending on the number of expected values, with 5% significance as the limit to reject the null hypothesis. Brain Metastasis Specific Survival (BMSS) i.e. time from diagnosis of BM to death were estimated by the Kaplan Meier estimator, and compared by the log rank test. Two patients who had not undergone surgery were removed from survival analyses. IBM SPSS Statistics for Windows v.25™ (https://hadoop.apache.org) was used for statistical calculations. Figures were created using Microsoft Excel 2016 ™ (https://office.microsoft.com/excel), and Adobe Illustrator 2020 ™ (https://adobe.com/products/illustrator).

### Ethical approval

The study has been conducted in accordance with the Declaration of Helsinki and the Sahlgrenska University Hospital Ethical Review Board, Gothenburg, Sweden approved the study (Swedish Ethical Review Authority, 460–09, T592-14), which also waived the need for informed consent due to the retrospective design. Approval for chart studies was approved by the departments of Surgery and Oncology at Sahlgrenska University Hospital, the department of Surgery at Näl Hospital, the department of Surgery at Halmstad Hospital, the department of Surgery at SKAS, and the department of surgery at SÄS.

## Results

**Figure 1:**
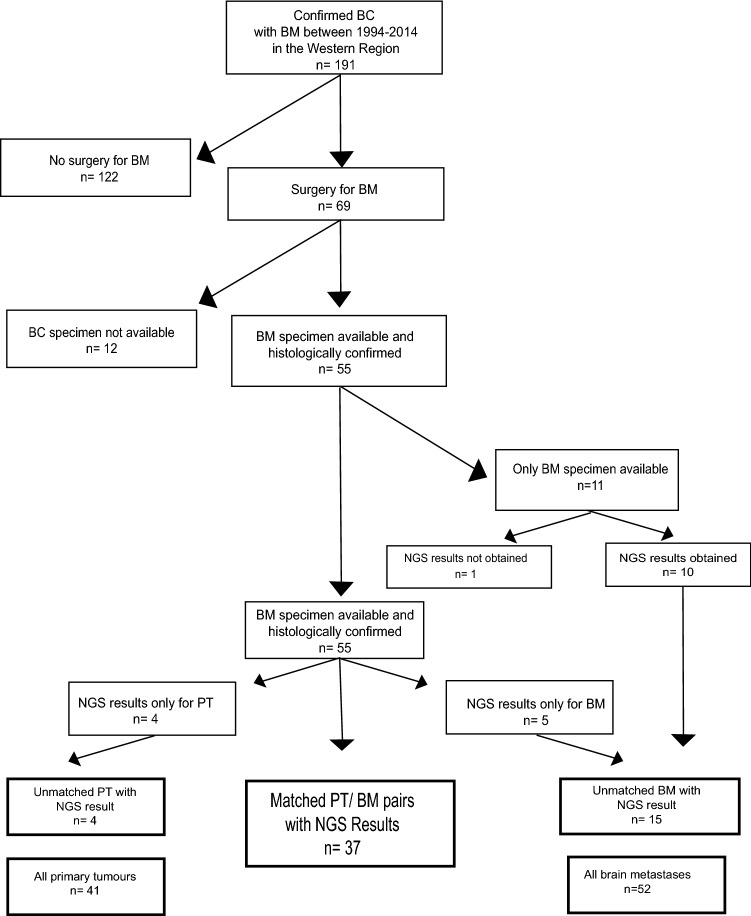
Consolidated Standards of Reporting Trials (CONSORT) diagram describing the identification process of the study population showing the final numbers of brain metastasis, primary breast tumors and matched pairs of brain metastasis and primary breast tumors.

After identifying 69 patients using diagnostic codes and charts, 46 patients remained with available matched PT and BM tissue. In addition 11 patients with only BM tissue were located. Thus, 57 BMs and 46 PTs were included in the study (Fig. 1). Of 57 patients with BM, 55 had undergone surgery (96%). The median age at time of BM diagnosis was 53 (29–75) years (clinical data is specified in Tables 1, 2). The 22 patients with extra-cranial disease (ED) prior to BM had a median time from ED to BM of 18.1 months (range 12 months).

**Table 1:** Clinical characteristics at primary breast cancer of the 41 patients with available primary tumor material.

	All (n = 46)	Luminal A-like (n = 6)	Luminal B-like (n = 6)	TNBC (n = 23)	HER2 + (n = 11)
**Age at diagnosis of BC (years)**					
Mean (standard deviation) (SD)	49 (9.9)	54 (10.1)	50 (8.2)	46 (9.5)	46 (9.3)
Median (min; max)	51 (32;72)	58 (40; 64)	51 (41; 60)	54 (32; 67)	45 (33; 55)
**Stage at diagnosis of BC**					
I	7 (15%)	0 (0%)	2 (33%)	2 (9%)	3 (27%)
II	13 (28%)	2 (33%)	2 (33%)	6 (26%)	3 (27%)
III	20 (44%)	3 (50%)	2 (33%)	11 (48%)	4 (36%)
IV	6 (13%)	1 (17%)	0	4 (17%)	1 (9%)
**Histological subtype**					
Ductal invasive	42 (91%)	6 (100%)	5 (83%)	22 (96%)	9 (82%)
Lobular invasive	3 (7%)	0 (0%)	0 (0%)	1 (4%)	2 (18%)
Other	1 (2%)	0 (0%)	1 (17%)	0 (0%)	0
**BRE grade**					
II	12 (26%)	2 (33%)	1 (20%	6 (26%)	3 (27%)
III	34 (74%)	4 (67%)	5 (80%)	17 (24%)	8 (73%)
**Brain metastasis free interval (months) (n = 46)**					
Mean (SD)	48 (52.26)	58 (45)	74 (21)	39 (38)	63 (34)
Median (min; max)	51 (11; 288)	49 (0; 113)	73 (48; 97)	33 (0; 196)	51 (34; 113)

BRE (Bloom–Richardson–Elston system, with 1–3 being grade I, BRE 4–7 grade II, and BRE 8–9 grade III.

**Table 2:** Clinical data of the 52 patients with available brain metastasis.

	All (n = 57)	Luminal B-like (n = 14)	TNBC (n = 26)	HER2+ (n = 17)
**Age at diagnosis of BM (years)**				
Mean (SD)	53 (10.9)	55 (10)	54 (10.7)	49 (11)
Median (min; max)	53 (29; 75)	55 (40; 70)	56 (29; 72)	44 (36; 75)
**Brain metastasis prior to other metastases**				
BM only	19 (33%)	5 (36%)	5 (19%)	9 (53%)
BM after extra cranial mets	22 (39%)	4 (28%)	13 (50%)	5 (29%)
BM before extra cranial mets	16 (28%)	5 (36%)	8 (31%)	3 (18%)
**Location of BM**				
Cerebrum only	33 (58%)	8 (57%)	16 (62%)	10 (59%)
Cerebellum only	15 (26%)	5 (36%)	8 (31%)	2 (12%)
Multiple locations	9 (16%)	1 (7%)	2 (7%)	5 (29%)
Meningeal carcinosis	5 (9%)		1 (4%)	4 (24%)
**Treatment for local control**				
None (material from autopsy)	2 (4%)		1 (4%)	1 (6%)
Surgical resection	16 (28%)	2 (14%)	10 (38%)	4 (24%)
Surgical resection followed	39 (68%)	12 (86%	15 (58%)	12 (70%)
By radiotherapy				
**Number of BM**				
1	48 (84%)	13 (93%)	23 (88%)	13 (76%)
2 or more	9 (16%)	1 (7%)	3 (12%)	4 (24%)

NGS results fulfilling sequencing quality criteria were obtained for 37 of the 46 matched BM/ PT pairs and from 10 of the 11 unmatched BMs. For nine matched pairs, sequencing results were obtained for either only the PT (n = 4) or the BM (n = 5). In total, NGS results from 41 PTs and 52 BMs including the 37 matched PT/BM pairs were obtained (Fig. 1). IHC subtype is reported for the matched 37 PT and BM only.

**Figure 2:**
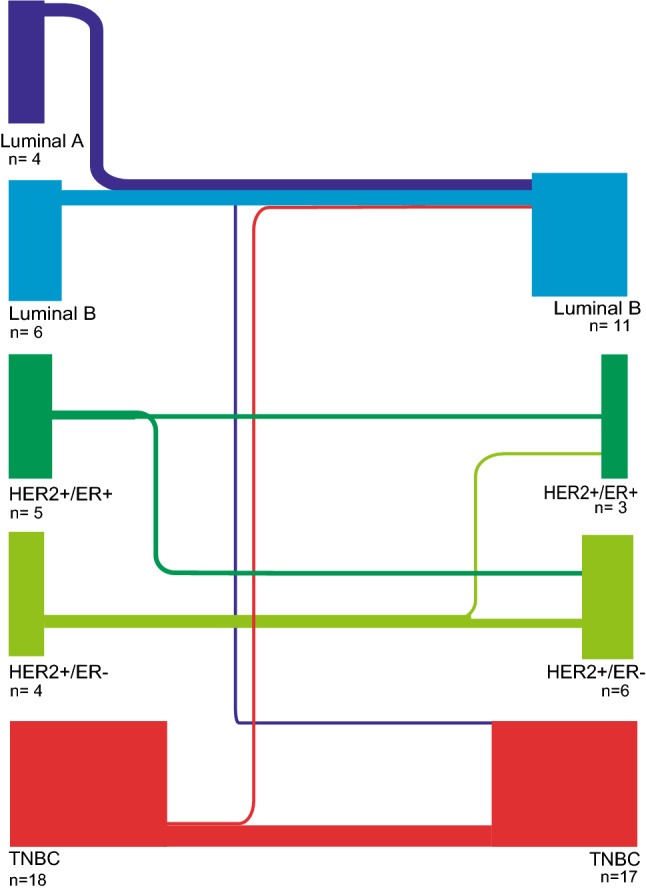
Breast cancer subtype according to expression of steroid receptors, HER2 and Ki67 in primary breast tumors and brain metastases. The thickness of the line represents the number of tumors that changed subgroup in respective group.

In the 37 matched BM and PT, receptor status was discordant in 8/37 matched pairs (22%). Three Luminal A-like tumors had an increase in Ki67 rendering them Luminal B-like, one Luminal A-like lost ER receptor expression rendering it TNBC. No Luminal B-like tumors altered expression. Two HER2/ER+ lost ER receptor, one HER2+/ER− gained ER receptor. One TNBC gained ER receptor expression (Fig. 2).

### Genetic profile of BM and PT

**Figure 3:**
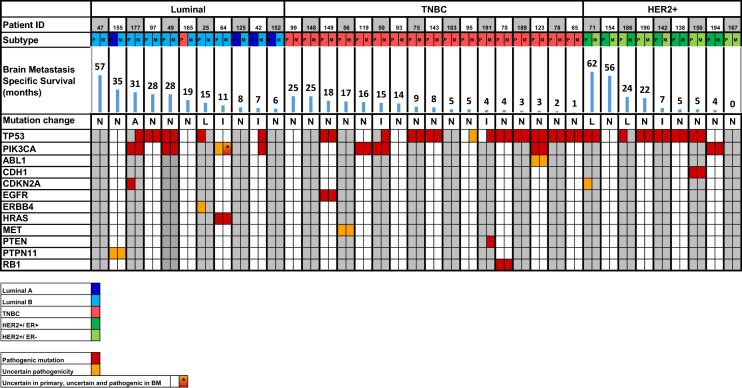
All matched pairs of brain metastasis and primary breast tumors color coded into subgroups with pathological and uncertain mutations shown. Brain metastasis specific survival (months) for each patient is shown in blue bars above the mutations.

Twenty-seven PTs (66%) and 32 BMs (62%) exhibited at least one pathogenic mutation (Fig. 3). A pathogenic *JAK2* p.V617F mutation in a patient with previously diagnosed myeloproliferative disease was excluded from further analysis. The most commonly mutated gene in the dataset was *TP53,* with a mutation frequency of 44% (20/42) in PTs and 44% (25/52) in BMs. *PIK3CA* mutations were the second most prevalent mutation, with a frequency of 20% (9/42) in PTs and 23% (13/52) in BMs. Of note, a *CDH1* mutation was present in a BM/ PT pair of lobular BC. Mutations in *EGFR*, *HRAS* and *RB1* were detected in single PT/BM pairs, as well as, mutations in *CDKN2A* and *PTEN* in single samples of PT or BM. All mutations may be studied closely in Supplementary Table S1.

### Mutational findings in matched pairs of BM and PT

In the matched pairs, similarly, the most prevalent mutation was in *TP53* in 43% (16/37)*.* Mutated *TP53* was found in three Luminal A- and B-like PTs and four paired BMs (27%/36%), eight TNBC PTs and ten paired BMs (47%/59%), and six HER2 positive PTs and five paired BMs (67%/55%). Mutations in *PIK3CA* was the second most prevalent mutation in this material. In matched PT/BM pairs, *PIK3CA* mutations were found in two PTs and four BMs of Luminal B-like (18%/36%); three PTs and BMs in TNBC (18%/18%), and in one matched case of HER2 positive tumors (11%) (Fig. 3, Table 3). The numerical differences of *TP53* and *PIK3CA* mutations in BC subgroups did not reach statistical significance (p = 0.228) (p = 0.552) respectively.

**Table 3:** *TP53* and *PIK3CA* mutations in primary breast cancer tumors and brain metastases with NGS results fulfilling sequencing quality criteria.

	All (n = 57)	Luminal B-like (n = 14)	TNBC (n = 26)	HER2 + (n = 17)
Mean (SD) brain metastasis specific survival (months)	17 (16.9)	28 (18.9)	9 (7.4)	21 (19.8)
Median (min; max) brain metastasis specific survival (months)	12.5 (0; 64)	28 (6; 64)	8 (1; 25)	13 (0; 62)
Mean (SD) overall survival (months)	69 (52.3)	98 (41)	57 (60.7)	66 (33.4)
Median (min; max) overall survival (months)	50 (10; 288)	91 (35; 177)	40 (10; 288)	96 (42; 107)

The bottom row shows the concordance in mutation status in the 37 matched brain metastasis and primary breast tumors.

### Mutational concordance in matched material

The mutational profile of the genes present in the targeted panel was discordant between the PTs and BMs in nine cases out of 37 (24%). The mutational differences displayed no discernible pattern. There were three cases of mutations present only in the PT, four with only mutations found in the BM, and one case of mutation in the same gene, but at another position BM. Of note, in two PT/BM pairs, a pathogenic *PIK3CA* mutation and five *TP53* mutations were detected only in the BM.

### Survival parameters

Patients with Luminal A-/B-like and HER2+/ER+ BC had the longest BMSS (Table 4) with a median of 28 months (6–64) and 34 months (7–62) respectively, whilst patients with HER2+/ER− and TNBC had BMSS of 11 months (0–46) and eight months (0–46) respectively (p = 0.0851). We found equal BMSS when patients with mutations in *PIK3CA* were compared to patients without *PIK3CA* mutation (p = 0.467). There was no difference in BMSS when patients with a *TP53* mutation were compared with patients without documented *TP53* mutation (p = 0.456).

**Table 4:** Survival parameters of the 57 patients with brain metastases analyzed with NGS.

**Primary tumors (n = 41)**					
*TP53*	20 (44%)	2 (40%)	2 (33%)	8 (40%)	4 (36%)
*PIK3CA*	9 (20%)	2 (40%)	2 (33%)	5 (22%)	5 (45%)

	All (n = 52)	Luminal A-like (n = 0)	Luminal B-like (14)	TNBC (n = 23)	HER2+ (n = 15)
**Brain metastases (n = 52)**					
*TP53*	25 (44%)		4 (29%)	13 (57%)	8 (53%)
*PIK3CA*	13 (23%)		5 (36%)	5 (22%)	3 (20%)

	All (n = 37)	Luminal A-like (n = 0)	Luminal B-like (11)	TNBC (n = 17)	HER2+ (n = 9)
Concordance of mutational status in matched pairs (n = 37)	29 (67%)		7 (64%)	14/17 (82%)	7/9 (78%)

## Discussion

Concordant with previous published data, we confirm that *TP53* and *PIK3CA* are the most common mutations in BCBM, as well as, the matched PTs, in to our very best knowledge so far one of the largest cohorts of genetically characterized BCBM^16–21^. Difficulties in accessing intra-cranial tissue samples have hampered development of systemic treatments that could have an effect in the treatment of patients with BMs. Our cohort, consists of 52 analyzed cases, and 37 cases with matched PT, all from one region in Sweden. The difficulty in obtaining tissue from the brain results in few tumors which limits the study when comparing BC subgroups or in survival analyses. Although the tissue material is partly old, with the first samples collected in 1994, sequencing results by NGS were successfully obtained in 90% of the BMs. At least one mutation, among 50 cancer driver genes, was present in 62% of the analyzed sample, similar to previous publications^16–21^. Concordant with these previous studies, we found *TP53* and *PIK3CA* as the most commonly mutated driver genes, both in the BMs and PTs. Of these two mutations, *PIK3CA* are considered targetable, whilst *TP53* mutations can be indirectly attacked through restoration of the transcriptional activity resulting in a functional wild-type TP53 protein^22,23^.

The drug alpelisib, is a PI3Kα-specific inhibitor available for patients with recurrent *PIK3CA* mutated HER2 negative Luminal A-/B-like BC^23^. Previous published data show that approximately 40% of ER positive PT harbor a *PIK3CA* mutation^24^. Two studies that compared *PIK3CA* mutations in PTs and BC metastases reported mutations in 33% of PTs and 30% of metastases and 45% of PTs and 53% in metastases respectively^18,25^. There were few or no patients with BMs in the above-mentioned studies. Our results in BCBM had a lower fraction of *PIK3CA* mutations, with 20% in PTs and 23% in the BMs. A recently published systematic review compared 164 BMs with its matched PT in 126 patients extracted from 13 studies and found *PIK3CA* mutations in 22% of the patients with BMs which in line with our results^26^. It might be hypothesized that *PIK3CA* mutations is lower in BMs compared to other metastatic sites, however, additional studies are required to adequately adress this question. As expected, we found the highest proportion of *PIK3CA* mutations in Luminal A- and B-like BC. Of note, in our material two out of eight pathogenic *PIK3CA* mutations and five *TP53* mutations were found in the BMs only, and not in the PT underlining the need for re-evaluation of metastatic tissue or possibly an added value by analysis of cell free DNA (cfDNA)^27^.

We found *PIK3CA* mutations as the second most common mutation after *TP53* in matched pairs of TNBC with 18% *PIK3CA* mutations all concordant in PTs and BMs. This is in line with previous published data in which *PIK3CA* mutations was the second most common mutation after *TP53*, especially in basal-like and androgen receptor expressing subtypes of TNBC^28–30^. The combined treatment of alpelisib and nab-paclitaxel is currently under investigation in pre-treated TNBC with either loss of PTEN expression or a *PIK3CA* mutation (NCT04251533).

The prognostic role of *PIK3CA* mutations in HER2+ BC has been extensively investigated. Mutations in *PIK3CA* have been associated with diminished effect of HER2 blocking therapy, both in the neo-adjuvant setting, and for recurrent BC in terms of lower pCR rates, and shorter survival respectively^31,32^. We found *PIK3CA* mutations in only 10% of the HER2+ cases, which is lower than the previously reported frequency of 20–40%^24^. This discrepancy may be due to the limited number of patients with HER2+ BC. The effect of alpelisib in HER2+ breast cancer is under investigation in one ongoing and one completed clinical trial (NCT02038010; NCT04208178).

Almost 50% of the PTs, out of which many exhibited TNBC subtype, in the present study population harbored a *TP53* mutation, in contrast to the 20–35% mutation prevalence reported in unselected primary BC^33^. The Cancer Genome Atlas reveals an enrichment of *TP53* mutations in basal-like and HER2 enriched BC^24^. Interestingly, previous data reveal that a high proportion of patients with a *TP53* mutation in the primary BC developed BM^34,35^. We lack data on the molecular subtypes in our material, but find an increased number of *TP53* mutations in TNBC and HER2/ER- BC. Enrichment of *TP53* mutations in BMs was not seen in the HER2 amplified subgroup, a finding that must be interpreted with caution due to the very small sample size of the HER2 group. Other detected mutations in our series, *CDH1*, *EGFR*, *HRAS, RB1, CDKN2A* and *PTEN* were rare, in general found in single or both samples from one patient.

Roughly 25% of the matched pairs changed IHC based BC subtype in the BMs. The most common change was from Luminal A-like in PT to other subtypes, in most cases Luminal B-like in BMs. This is in accordance with previous results in which 219 patients showed a 36% overall discordance with the most common change in form of loss of PgR^36^. In a review pooling a total of 3384 matched pairs of BC and metastases from all organs, BM showed a discordant median rate of 22% compared to 45% in liver metastases and 16% in lymph node metastasis^10^. TNBC was the most stable group with less than 10% showing a gain in ER (one case) and HER2 (one case).

A limitation of our study is that genetic profiling was performed using a relatively small NGS panel of 50 genes. Although the panel provides broad coverage of important cancer driver genes, more comprehensive sequencing might provide a more granular view of mutation discordancy between PTs and BMs. Still, we believe that the relatively large number of matched PT and BM add to the knowledge about the biology of BMs, and changes in the mutational landscape of actionable genes during the metastatic process.

In conclusion, we confirm mutations in *TP53* and *PIK3CA* to be common in both primary breast tumors and BMs but the proportion varied depending on the subgroup. Mutation pattern, as well as IHC based subtypes were discordant in approximately 25% of the patients underlining that re-biopsy at disease progression may be warranted. In this context, analysis of cfDNA may be a fruitful avenue in patients with BMs, placing hope in promising results with liquid biopsy from CSF^27,37,38^.

## Supplementary Information

Supplementary Table S1.

## Data Availability

All pathological and uncertain mutations are added as a Supplemental Table. Clinical data is not included as this would allow for possibly identifying individuals.
